# More Than Needles: The Importance of Explanations and Self-Care Advice in Treating Primary Dysmenorrhea with Acupuncture

**DOI:** 10.1155/2016/3467067

**Published:** 2016-05-08

**Authors:** Michael Armour, Hannah G. Dahlen, Caroline A. Smith

**Affiliations:** ^1^The National Institute of Complementary Medicine, Western Sydney University, Locked Bag 1797, Penrith, NSW 2751, Australia; ^2^School of Nursing and Midwifery, Western Sydney University, Locked Bag 1797, Penrith, NSW 2751, Australia

## Abstract

*Background.* Primary dysmenorrhea is a common gynaecological condition. Traditional Chinese medicine (TCM) acupuncturists commonly treat primary dysmenorrhea and dispense specific self-care advice for this condition. The impact of self-care advice on primary dysmenorrhea is unknown.* Methods.* 19 TCM acupuncture practitioners from New Zealand or Australia and 12 New Zealand women who had recently undergone acupuncture treatment for primary dysmenorrhea as part of a randomised controlled trial participated in this qualitative, pragmatic study. Focus groups and semistructured interviews were used to collect data. These were recorded, transcribed, and analysed using thematic analysis.* Results.* The overarching theme was that an acupuncture treatment consisted of “more than needles” for both practitioners and participants. Practitioners and participants both discussed the partnership they engaged in during treatment, based on openness and trust. Women felt that the TCM self-care advice was related to positive outcomes for their dysmenorrhea and increased their feelings of control over their menstrual symptoms.* Conclusions.* Most of the women in this study found improved symptom control and reduced pain. A contributing factor for these improvements may be an increased internal health locus of control and an increase in self-efficacy resulting from the self-care advice given during the clinical trial.

## 1. Introduction

Primary dysmenorrhea is defined as painful uterine cramps of menstrual origin in the absence of any organic cause and is most common in women under the age of 25, with pain usually starting within three years of menarche [1, 2]. A recent review of high-quality studies found prevalence rates to range from 16 to 81% [3]. Primary dysmenorrhea is responsible for a decrease in quality of life [4–6], absenteeism from work or school [7], reduced participation in sport and social activities [8], altered pain perception [9], and sleeping problems [10]. Despite this, women underreport dysmenorrhea during medical consultations [11] and believe that period pain is a normal part of the menstrual cycle [8, 12, 13] with most women using over-the-counter (OTC) analgesic medication and self-management strategies to treat their period pain rather than seeking medical advice [4, 5, 8, 12, 14–20]. Even with analgesic medication, many women get unsatisfactory relief of their menstrual pain [21, 22]. Lack of satisfactory pain relief and effective medical interventions in primary dysmenorrhea leads to an uptake of self-care strategies by women [23].

Complementary and alternative medicine (CAM) usage is often a significant component of self-care [23, 24]. Acupuncture has become a popular CAM therapy for a variety of women's reproductive health conditions [25, 26]. Recent systematic reviews show promise in reducing menstrual pain via acupuncture [27, 28]. European acupuncturists believe that acupuncture shows significant promise in treating gynaecological conditions [26], and patients present to acupuncturists in the community with gynaecological complaints [29] including primary dysmenorrhea [25].

An important component of traditional Chinese medicine (TCM) acupuncture is the dispensing of self-care advice. Self-care is a broad concept incorporating efforts by the patient towards reduction of symptoms, maintenance of good health, and prevention of poor health [30]. Surveys show that self-care advice is commonly given as part of acupuncture treatment [25, 26, 29, 31]. The importance of TCM self-care advice has been discussed by other acupuncture practitioners when treating a variety of conditions, including musculoskeletal conditions [32, 33], a variety of internal medicine disorders [34], and depression [35, 36]; however, its role with the management of primary dysmenorrhea is unclear.

Common components of self-care talk in acupuncture consultations cover diet, over-the-counter medications, physical activity, rest and relaxation, practice of related therapies (i.e., Qi Gong), and protection from the elements [37]. Acupuncture practitioners view this advice as “empowering” rather than “instructional” and an important factor in allowing patients to move from passive to active roles with regard to their health [33]. Feelings of empowerment and increased agency have been expressed by patients following treatment [38–41]. This self-care advice can allow patients to come to a different understanding of their current condition [32, 37, 39]. Therefore, it is plausible that self-care advice is at least partially responsible for the changes in attitudes related to the presenting complaint that is often seen in acupuncture patients [39]. Despite practitioners' belief that adherence to this advice gives better outcomes [33], there have been no studies examining patient compliance to this self-care advice and its relationship to therapeutic outcomes.

The aim of this study is to examine the impact of different components of TCM acupuncture treatment on the outcomes of primary dysmenorrhea from the perspective of practitioners and women who have had acupuncture treatment for primary dysmenorrhea.

## 2. Methods

This study was carried out using a qualitative, pragmatic methodology and formed part of a study examining the effect of changing the dosage of acupuncture on the symptoms of primary dysmenorrhea.

### 2.1. Practitioner Background

MA, the primary investigator, is also a practicing TCM acupuncturist who treats women with period pain in his practice. Due to the involvement of the researcher in this area, reflexive memos were kept and both insights from these memos and all transcripts were discussed with CS and HD, both of whom are not practicing acupuncturists. In addition, a postdoctoral researcher who was experienced in acupuncture but unaffiliated with the research project recorded the focus groups and was involved in discussion regarding the need to clarify or alter questions after the first focus group was undertaken.

### 2.2. Sampling: Practitioners

A previous survey of Australian and New Zealand acupuncturists [25] included an invitation to participate in further research if participants considered themselves “very experienced” in treating period pain and were in clinical practice for more than 20 hours per week. Purposive sampling was used based on the assumption that those with significant experience in women's health would yield significant information and provide unique perspectives based on their experience in the field [42]. Sixty-two practitioners expressed an interest in participating in the focus groups and/or semistructured interviews (SSIs). Ten acupuncture practitioners participated in two focus groups of five participants in each group in Auckland and Wellington, New Zealand. Nine SSIs were carried out via Skype for Australian practitioners. Both focus groups and the practitioner SSIs were performed by MA.  Table 1 shows the demographics of the participating practitioners. One female practitioner from New Zealand with > 10-year experience who was involved in one of the focus groups delivered a number of treatments during the RCT; however, her involvement in the RCT was not discussed until after practitioner data collection was complete.

### 2.3. Sampling: Study Participants

Women who participated in a randomised controlled trial (RCT) on the effectiveness of TCM acupuncture on primary dysmenorrhea (manuscript under preparation) were offered the opportunity to participate in the interviews. Two trial sites were represented, Auckland and Wellington, New Zealand. The inclusion criteria for the RCT were women with suspected or confirmed primary dysmenorrhea as defined by the following: age between 18 and 45 years, a history of period pain beginning before the age of 18 or period pain beginning after the age of 18, with gynaecological investigations by laparoscopy and/or ultrasound scan showing no evidence of secondary dysmenorrhea, pain greater than or equal to 3 out of 10 on a numeric rating scale (NRS) during the first three days of menses for at least two of the past three menstrual cycles, and regular menstrual cycles (28 ± 7 days) for the last three months. Exclusion criteria for the RCT were a previous diagnosis of endometriosis or secondary dysmenorrhea, abdominal surgery in the previous three months, injectable or implant contraceptives (Depo Provera, Jadelle, and Mirena) within the last three months, oral contraceptive usage started less than three months prior to enrolment, chronic pain conditions (>14 days per month with pain), and neuropathic pain secondary to surgery or sterilisation. The inclusion/exclusion criteria were chosen to try and replicate the types of patient who were likely to present for treatment in the community.

Purposive sampling was then used on the sample of women who consented to interviews based on the trial site and the self-rated effectiveness score in the exit questionnaire. The number of women from each site was chosen to represent the 40%/60% geographical split in participants and to ensure each site was represented in proportion to its contribution to overall results in the RCT. Twelve women with a mean age of 30.9 years were interviewed in the order they finished the trial (four from Wellington and eight from Auckland). The effectiveness score was a self-rated 0–10 NRS in response to the question “how effective did you find the acupuncture treatment for your period pain?” with 0 being no change and 10 being significant improvement. Women were classified as responders if they rated the trial as having an effectiveness score of >5/10, while those rating ≤5/10 were considered to be nonresponders.  Table 2 provides details of each woman interviewed. The following list is the topic guide used for focus groups and semistructured interviews:

- How effective have you found acupuncture treatment for period pain to be in your practice?
- In your treatment of dysmenorrhea do you have a typical treatment frequency or pattern of treatment that you like to use?
- In your experience how many treatments are usually necessary to see a significant improvement in dysmenorrhea?
- What are the key improvements/outcomes you are looking for in treating period pain?
- Electroacupuncture (EA) is often used for pain in musculoskeletal disorders but our survey shows it does not appear to be used a lot in painful menses. Why do you or do not you use electroacupuncture?
- If you do use EA in your clinic, what kind of frequency and duration do you usually use?
- When using manual acupuncture for period pain what kind of stimulation do you use?
- In our survey we found that a lot of practitioners give lifestyle and diet advice for their period pain patients. How important do you think this is in the outcome of your treatments?
- Do you personalize the advice to each woman or do you tend to give similar advice to most women?

### 2.4. Practitioner Interviews and Focus Groups

A broad focus on acupuncture for the treatment of primary dysmenorrhea was the starting point, and subsequent subtopics relating to this overarching idea were explored. An interview topic guide containing open-ended questions was used to ensure that all specific areas of interest were covered (see the previous list). Focus groups lasted approximately 90 minutes while the SSI lasted between 30 and 45 minutes, both of which were digitally recorded. Despite differences in both geography and data collection method, the themes that emerged from both focus groups and SSIs were essentially identical.

### 2.5. Participant Interviews

A written question guide was used in these interviews and participants were asked to share their experience of receiving acupuncture for period pain. Participants were encouraged to explain in their own words the experience in the trial and if it had changed their perceptions of health and wellbeing. A specific focus was placed on the uptake of diet and lifestyle advice, due to the importance of these contributing to a positive treatment outcome by the study acupuncturists. The following list includes interview schedule used for interviews with women who participated in the trial:

- How was your experience of participating in the trial?
- What was your experience with acupuncture before being in the trial?
- You were in the XX group, this meant you had treatment every YY and had (electro-/manual) stimulation on the needles. How did you find being in this group?
- As part of your treatment, your practitioner should have given you a sheet on lifestyle and self-care advice. Did you use the self-care advice they gave you?
- What are your views on whether period pain is normal?
- Before participating in this study, whom had you talked to or got advice from about your period pain?
- What did you think of explanations that your acupuncturist gave you about the cause of your period pain?
- Did your view of your period and the symptoms change between the start and end of the trial?
- Looking back on your time in the trial what did you think of your relationship with your acupuncturist?

The interviews were conducted via Skype by either an independent researcher with a background in acupuncture and experience in conducting qualitative interviews for those women who located in Auckland or the primary investigator (MA) for those women who were located in Wellington. The primary investigator (MA) only treated women in Auckland and did not interview any participant where he had administered treatment. Interviews were conducted over approximately 30–45 minutes and were recorded on the interviewer's computer before professional transcription was carried out.

### 2.6. Data Analysis

**Figure 1:**
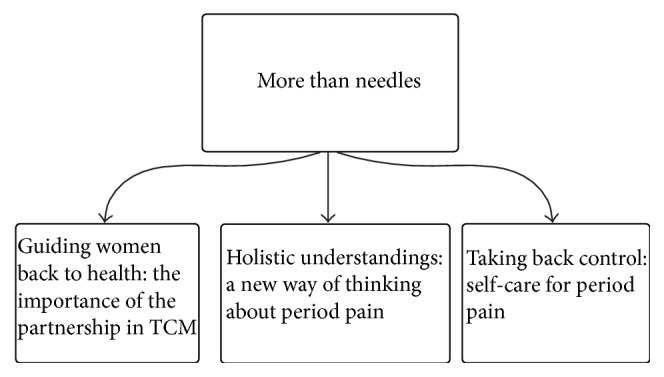
Themes and subthemes from the integrated datasets.

Both focus groups and SSIs were digitally recorded and professionally transcribed into MS Word format documents. NVivo (NVIVO 10) was then used to undertake line-by-line coding. Thematic analysis [43] was used to report themes in the data after each dataset was transcribed. Reflexive memos were used and negative cases were examined. After each interview, preliminary thematic analysis was carried out using the constant comparative method described by Bowen [44] to determine if any new codes or themes were present. Saturation in this sample was noted after 19 practitioners and 12 study participants, with no new codes being applied to the data and no new themes emerging from data at this point. Triangulation was used to integrate the findings of the practitioner and participant data [45]. Triangulation occurred after both datasets had been analysed separately. MA, CS, and HD then listed the findings of each dataset in a table looking for findings that agree (convergence), offered complementary information, or contradicted other findings (dissonance).  Figure 1 outlines the themes that emerged from triangulation of both datasets. Two authors (HD and CS) provided critical feedback and assisted with theme development and refinement. Any disagreements were resolved by discussion.

### 2.7. Presentation in the Text

Practitioners were allocating names based on their current practice location (Auckland, Wellington, or Sydney) with the training location added if they had trained in China. Women who participated in the interviews were given pseudonyms.

### 2.8. Ethical Approval

The practitioner research was approved by the University of Western Sydney Ethics Committee (H9866), while that involving participants was approved by both the UWS Human Ethics Committee (H10082) and the HDEC New Zealand (13/CEN/60). Written informed consent was obtained from all participants.

## 3. Findings

A key, overarching theme that emerged across both practitioners and women was that acupuncture treatment was “more than needles.” Acupuncture practitioners in the focus groups emphasised the unique nature of the TCM theoretical framework that allowed them to practice from a holistic outlook. Acupuncturists were very clear that an acupuncture treatment encompassed much more than just the insertion of needles and that other components of treatment, such as TCM explanations of health and disease, moxibustion, and self-care advice, were also very important contributors to the therapeutic outcome and were intrinsic components of their treatment. Women confirmed the importance of the TCM explanations of menstrual physiology and specifically self-care advice, as key to increasing their self-efficacy. Women discussed how the context in which these explanations and advice were delivered, in a nonjudgmental and supportive environment, was different to their previous experiences with general practitioners and how this relationship with their acupuncture practitioners impacted their ability to understand their menstrual cycle better and implement the self-care advice given. Three key themes were nested under the overarching theme: “*guiding women back to health: the importance of the partnership in TCM*,” “*holistic understandings: a new way of thinking about period pain*,” and “*taking back control: self-care for period pain*.”

### 3.1. Guiding Women Back to Health: The Importance of the Partnership in TCM

This theme discusses the relationship that participants had in the study with their practitioner and how it was this relationship that provided the foundation of trust and mutual understanding that enabled women to gain new understandings and better implement self-care advice.

*So I look at it as like a partnership, that I'm here to guide them on their journey to get well. (Wellington Practitioner 5)*

Women who had been in the study stressed the importance of the partnership in the areas of trust and understanding. Participants commented on how they felt like they could be open with their practitioners, trust them, and discuss issues with them.

*Yeah, yeah. Somehow I felt so comfortable, I could express myself freely and feel supported whenever I said whatever was going on, whatever I just be honest and open and yeah…. (Betty)*

Participants mentioned that they felt “safe” with their practitioners, and this safe environment was an important part of being able to trust them, since talking about periods was often a private topic and made them feel exposed.

*I just felt I could go and speak openly and that was two-way as well. So he would talk to me about a thing and it wasn't inappropriate and feel inappropriate or uncomfortable. Just felt safe and supportive and nurturing and just funny, just light hearted you know instead of just feel better afterwards for just sort of talking to someone who seem to be supportive and not judgmental and just kind of relate to what I would say as well. (Betty)*

Practitioners relinquished the role of “expert” and repeatedly mentioned the idea of empowering women to become their own “experts” in the field of their own health, by dispensing their accumulated knowledge over time and thereby further reducing the imbalance power in the relationship.

*I guess in my clinical space I like to maximise the power of the patient and I don't like to take an expert role. (Sydney Practitioner 1)*

### 3.2. Holistic Understandings: A New Way of Thinking about Period Pain

This theme examines the transformation in women's understanding of period pain, from something “normal” and almost symbiotic with the period to an understanding of period pain as being a holistic condition that can be understood and managed by women themselves.

*When you say to people, you don't need to be having this kind of pain. It's not actually normal. They [are] kind of – it's a bit like, oh, there's a whole other life out there. So I think it's part of how society views periods and that a bit of pain is – or pain is expected. So it's educating them about that as well, and that spreads the word. (Wellington Practitioner 2)*

Women commented that period pain, in their mind, was just part of “being a woman” and it was something that women had to put up with as an integral part of womanhood; that is, if you are a woman then you get period pain.

*Yeah it's kind of just … I don't know … it sounds weird … a reminder or right of passage, it's just like you know … because you are a woman, this is what you get. (Sarah)*

Most women felt that their attitude around seeking treatment for period pain was formed by the dominant narrative; that is, it was normal to have this pain; therefore, there is no point in trying to treat it.

*I'm not really sure, I suspect part of it was the whole attitude; well it's not something that needs treating because it's normal. So because of that idea of being normal it's in my head, it didn't occur to me that … therefore it's normal and therefore you don't need to do anything about it I guess. (Elizabeth)*

Practitioners also often found that women would not mention painful periods unless they are specifically asked, as period pain was often not considered a health condition or even a symptom worth mentioning.

*Generally what I find is that women are so conditioned to the fact that periods are painful, that they don't actually think of it as being a symptom, and when you ask them about it, they say oh but that's normal for me. (Auckland Practitioner 1)*

Quite a few of the women had discussed their period pain with friends and family but had been given the impression that it was something that was just normal and was not really a medical problem and this seemed particularly the case when other close family members had period pain.

*Yeah I just sort of talked to mom and she gets them quite bad as well, so I just thought it must be normal. (Leslie)*

Practitioners felt that an explanation of a “normal” period according to TCM was well received by their patients, and women found that it exciting or were surprised that they did not have to necessarily have pain every period.

*Well certainly they are excited by the possibility that they don't have to have pain and that they don't have to put up with pain and that, in fact, it's not what we regard as normal in Chinese medicine. (Sydney Practitioner 4)*

Women often found that when discussing their period pain with their acupuncturist, it was quite a surprise to them that TCM did not consider period pain normal or inevitable and that they could have periods with less or no pain.

*Yeah it was and as I say, because I'm a health professional myself and it was only when [acupuncturist] and I started discussing this and whether I would be eligible for the trial and just him saying “well actually it's not necessarily something that should be normal or normalised” and it really kind of turned my head around if you like “Huh? I really hadn't thought about that but actually you're right!” (Elizabeth)*

Several of the women made mention of how once they knew it was not normal, it gave them hope that they did not need to “settle” for the same kind of pain each month.

*I think when I had my first session with [acupuncturist], she said quite a lot of women don't get any pain at all or just very gentle pains, she's like this is not something that you have to live with if you can treat it, so it kind of made me realise that maybe that wasn't normal and I should not be settling for it and I should be trying to improve it. (Teresa)*

How the period and menstrual cycle were explained by the acupuncturists, in the context of a TCM framework, gave women a different “reality” or context in which they understand their period pain.

*It was like a light bulb going on, you know, a new reality, and I quite like it when you know you are into something that kind of challenges your preconceptions or your idea for a while and it's in a positive way. (Kate)*

### 3.3. Taking Back Control: Self-Care for Period Pain

A major theme of taking back control of the menstrual cycle, specifically around reducing pain and other symptoms, first emerged in practitioner interviews and focus groups, where practitioners discussed empowering women via diet and lifestyle advice to be able to treat themselves. Self-care advice based on TCM theory was seen as the modality that could enable women to self-treat effectively.

*Diet and lifestyle is the key. I am very much of the acupuncturist preventive medicine persuasion and acupuncture is part of the philosophy of Chinese medicine and a lifestyle philosophy, so I do a lot of diet and lifestyle advice. (Sydney Practitioner 4)*

Most of the practitioners emphasised the importance of the thermal nature of food choices in TCM, with food being “hot,” “cold,” or “neutral.” There was an emphasis for most women on avoiding cold food due to the perception that many women with period pain had either an excess of cold or a lack of warmth in their bodies, which contributed directly to their period pain.

*I normally try and say to them, these are some dietary advice I like to give you because you've got a cold uterus, right, so it's best for you to eat warming foods and there's these other reasons why […] because it's all about keeping that area nice and warm and things flowing. (Wellington Practitioner 5)*

An important point was that women could share some of the responsibility for their wellbeing; that is, acknowledging previous diet and lifestyle choices contributed to the current disharmony was important as this meant that it was not solely the practitioners' responsibility to “cure” them.

*I pretty much give advice to everyone, and just simply, for them to be able to see that their lifestyle and whatever they've been doing in their whole life, has been leading to this point, which is in some state of disharmony, and if I can just highlight even just a couple of factors, what I think is leading them to this way, and they change that, they could get better theoretically by themselves over time. (Auckland Practitioner 3)*

Practitioners also were quite certain of the outcome of treatment if the self-care advice was not followed or changes to the patient's lifestyle were not made. This was due to the fact that practitioners felt that whatever the patients had done in the past, this was a major contributing factor to the imbalance or disharmony they were experiencing.

*… you've got some dietary and lifestyle stuff with all of them actually, it has to kind of go hand in hand and my thing is usually that if you don't change what you're doing you'll be back seeing me in 12 months' time because your lifestyle will reproduce these patterns. So I think that's important for lasting change. (Sydney Practitioner 3)*

Most women were surprised that changing their diet or choosing to go to bed earlier would have an impact on something like period pain and that this could be done simply by them.

*Yes. Something I found surprising. For example, I guess I didn't realise that period pain was so holistic and so in relation to a whole lot of different things, for example, like sleep or like reduction of caffeine. I mean these, you know, these sort of general common-sense things in there. I guess learning about period pain is as a response to not looking after yourself totally in certain areas. So, yeah just you know little things like certain diet changes, the way I eat and gentle exercises. (Betty)*

One of the benefits of the explanations of the TCM understanding of cause and effect was that women were more aware of the impact that their lifestyle and diet choices were having on their symptoms. Women often felt that once they knew what to look for, they could more easily see the impact their choices made on their menstrual symptoms.

* Yeah for sure, you know like the cold thing, like that was in my mind when I was getting sick of my cold coconut water that kind of stuff so I understood that there was an impact on what I was doing and how I would actually feel as a result so that was quite cool, yeah. (Hannah)*

Even participants who did not get total relief from their pain symptoms during the trial still found that the advice given to them about their periods and period pain is useful, as it provided them with a different explanation of what was causing their symptoms. In tandem with the lifestyle and diet advice they received, these explanations often made them feel empowered, as they felt like they had some agency over their bodies.

*Well yeah I mean now I feel like I am empowered to be able to do something about it that's more natural rather than taking any drugs and so yeah I feel like its separate from the pain in a way and I don't have to put up with the pain which before I would have. (Kate)*

## 4. Discussion

The findings from this study show that both acupuncture practitioners and women who have received acupuncture treatment for primary dysmenorrhea feel that their treatment encompasses more than just needles. Three key features of acupuncture treatments were discussed: how the TCM explanations for a normal menstrual cycle changed women's perception of what a normal menstrual cycle should be, how the self-care and diet advice given during the treatment sessions empowered women to better manage their own menstrual symptoms, and finally how the trust and support practitioners provided as part of this partnership allowed women to feel heard and understood and this in turn allowed them to feel confident implementing those lifestyle changes.

The partnership model of treatment was the underlying support that the rest of the acupuncture intervention was built on. Practitioners in our focus groups emphasised the importance of listening to, understanding, and educating the women. TCM acupuncturists tend to “coconstruct” their advice and explanations, which involves the practitioner and client shaping explanations and advice given by both and initiating and interacting in various kinds of “talk” [37]. This allows practitioners to understand the “life-world” of the client and deliver explanations, advice, and treatment that were appropriate and achievable for each individual. Consultations also included social talk, which helps practitioners build rapport and empathy with clients [34, 37]. Empathy from practitioners has been shown to increase enablement and is linked to positive treatment outcomes through the improvement of self-efficacy [32, 46]. Improved self-efficacy is also linked to increased optimism of future improvements [47]. An important issue that acupuncture practitioners highlighted in the focus groups was that they listened to women; practitioners thought that women had not really been listened to and that their menstrual experience was discounted due to the superior medical knowledge of doctors. Women in the interviews confirmed this, explaining how they felt like their symptoms were being taken seriously by study practitioners, rather than disregarding the impact that pain had on their menstrual experience, as often happens in consultations with their general practitioner [48]. Women commented that they finally felt “heard” by their study practitioners, who did not downplay their experiences, and that they felt “understood” by their practitioner. Being heard and understood is an important component of what Engel (1988) terms the patients “double need”: one to know and understand and one to be known and understood [49].

Practitioners who participated in the interviews and focus groups emphasised the fact that they often found that women did not reveal or list period pain as a symptom when presenting in their private clinics because it was thought of as “normal.” A possible reason put forward by our acupuncturists for this is the historical belief that it was at least partially just “part of being a woman” [15, 50, 51]. Practitioners in our focus groups commented on how they often noticed that women were surprised by the concept of having reduced pain or a painless period; that is, this was a concept they either did not believe was possible or had never considered. This is not uncommon in women who have had dysmenorrhea onset soon after menarche [52], as was the case with our participants, where most had dysmenorrhea onset approximately two years after menarche. Women in our interviews confirmed the practitioners' observation, commenting that periods and pain were almost symbiotic, and so the idea of a period without pain was something they had not considered possible. The concept of having to endure menstrual symptoms as an integral part of female life is common amongst women with dysmenorrhea [12, 53–55]. Women, almost without exception, expressed that, prior to enrolling in the trial, they thought that period pain was, to at least a large extent, a normal part of being a woman, with one participant echoing the historic idea “because you are a woman, this is what you get.” Despite an understanding of the physiological basis of primary dysmenorrhea, over the last 30 years attitudes by women on the normality of dysmenorrhea do not seem to have changed much [51].

All women commented on how TCM explanations, given as part of their acupuncture consultations during the RCT, were an important turning point in how they viewed their period pain; it changed from an inevitable “part of being a woman” to something that could be modified so they no longer had to “put up with it.” Explanations and education form the second component of Engel's “double need”: the patients need to know and to understand their condition [49]. Patients often feel that in orthodox medical consultations they do not get sufficient explanations for what is causing their condition, something that they feel they do obtain from CAM therapies [56]. Women expressed how the explanations given by their acupuncturists were framed in terms that they could understand, using a mixture of lay concepts (such as “energy”), the use of specific TCM concepts, and/or analogies depending on the individual circumstances. Women often did not remember all the TCM explanations, but they rather remembered how their practitioner distilled it into something that made sense to them. These kinds of credible explanations for symptoms are an important part of how patients attribute expertise to practitioners outside the mainstream medical system [57].

Women commented that the alternative health paradigm provided by TCM leads to a new, more “hopeful” idea about what constituted a normal period, with expectations of being in less pain over time as a result of being able to manage their symptoms via lifestyle choices. Hope and anticipation of improvement are an important aspect of treatment and can influence physiological processes, especially pain [58]. Period pain was now often reinterpreted to be a “holistic” issue, with physical and emotional contributors, rather than a specific gynaecological disease that needed to be treated.

Women in our study discussed how self-care was the most common way with which they dealt with their period pain, and each woman had tried a number of strategies to find the optimal combination. Practitioners discussed how self-care advice would empower patients to take more responsibility for their own health and be able to, in the words of one practitioner, “get themselves better over time,” rather than having to be passive consumers of health care that a practitioner must “fix.” Similar themes around increasing agency via self-care advice and thus allowing patients to “look after themselves” appear regularly in interviews with acupuncture practitioners treating a variety of disorders [33, 36, 59, 60], suggesting that this empowerment of patients via self-care is a common element of acupuncture treatment. Interview participants felt that the advice they received from their acupuncture practitioners empowered them to be able to take more control over their menstrual cycle.

A plausible explanation for the empowering nature of the self-care advice is that it increases women's internal health locus of control (HloC). Health locus of control is a measure of how much control individuals feel that they can exert over their own health, as opposed to their health being strongly influenced by either luck and/or chance or powerful external entities, such as God or a doctor [61]. Previous research has found that CAM users, or those that rate CAM use highly, have a higher internal HloC than those who use predominantly biomedicine [62, 63]. Despite the importance placed by practitioners on self-care advice for positive treatment outcomes, we did not find any evidence from our participant interviews that women who reported less use of diet and lifestyle advice during the treatment period or during follow-up felt like they achieved less pain relief.

The importance of this research is twofold. Firstly self-care advice delivered during acupuncture consultations appears to contribute significantly towards therapeutic outcomes, at least in part due to increasing women's internal HloC, and therefore practitioners should be encouraged to deliver this advice during acupuncture consultation sessions. Secondly, research on acupuncture which does not include self-care advice may be underestimating the therapeutic effect of acupuncture as delivered in the community.

The strength of this research is the use of triangulation, which allowed practitioners and participants views on the same specific topic to be integrated and contrasting viewpoints examined. A cross section of women who participated in the RCT were interviewed, both those that responded and those that did not respond to the acupuncture treatment, to ensure that self-care implementation did not differ amongst these two groups. The weakness of this research is that the women interviewed had been in a trial where self-care advice was delivered as part of the treatment protocol and may not reflect the variety of self-care advice delivered in community clinical practice. The participants, both practitioner and women with dysmenorrhea, were from New Zealand or Australia; therefore, our findings may not be generalizable to other populations, especially if there are significant cultural differences in how acupuncture treatments are delivered. The focus of purposeful sampling meant that there was an imbalance in the acupuncture groups represented in this study, with more manual acupuncture participants being represented. However, there was a nonsignificance between group differences in self-reported effectiveness rates at the conclusion of the RCT. Therefore, the differences in responder rates in Table 2 do not represent responder rates in the RCT between groups. The median self-reported effectiveness rate at trial end was eight; this high responder rate in the trial made recruitment of women classed as nonresponders more difficult due to the small number of women who fell into this category. However, we actively sought out nonresponders to ensure reporting was as balanced as possible. Our sample size was small; however, saturation was achieved and many themes are supported by studies on using acupuncture for other conditions.

## 5. Conclusion

TCM acupuncture treatment was considered to be more than just the insertion of acupuncture needles by both practitioners and women who received acupuncture treatment. Women reinterpreted their concept of what a normal period should be like, and the idea of a period free from pain as the new normal gave them hope. Self-care advice, delivered through the TCM framework, was an important component in providing a more hopeful outlook for future periods amongst women. The partnership between the practitioner and women provided the support and trust necessary to implement the diet and lifestyle changes suggested. Women who suffer from primary dysmenorrhea may achieve symptom relief and improved quality of life with TCM acupuncture via changes in self-efficacy and improving their internal health locus of control.

## Figures and Tables

**Table 1:** Demographics of practitioners in focus groups and interviews.

Location	New Zealand (NZ) *n* = 10	Australia (AU) *n* = 9
*Age (y)*	38	40

*Gender *		
Male	2	1
Female	8	8

*Training location*		
NZ or AU	9	4
USA	1	0
China	0	5

*Years in practice *		
<5 years	1	0
5–10 years	5	2
>10 years	4	7

Age is the mean age of each location group.

**Table 2:** Characteristics of interview participants.

	Overall (*n* = 12)	Group 1 (*n* = 4) Three weekly manual acupuncture treatments each cycle	Group 2 (*n* = 3) Three weekly electroacupuncture treatments each cycle	Group 3 (*n* = 4) Three manual acupuncture treatments in the seven days prior to menses each cycle	Group 4 (*n* = 1) Three electroacupuncture treatments in the seven days prior to menses each cycle
*Age (y)*	30.9	32.1	29.8	32.5	29

*Responder *					
Responder	8	4	1	3	0
Nonresponder	4	0	2	1	1

*Trial site*					
Auckland	8	3	2	3	0
Wellington	4	1	1	1	1

*Previous use of acupuncture*					
Yes	7	2	2	2	1
No	5	2	1	2	0

*Adverse event reported during trial*					
Yes	0	0	0	0	0
No	12	4	3	4	1

*Expectation of benefit from acupuncture*					
Unsure	4	2	1	1	0
Probably help	7	2	2	3	0
Definitely help	1	0	0	0	1

Group is the group allocated to the women in the randomised controlled trial. All groups received the same number of individualised TCM acupuncture treatments (12) over three menstrual cycles. All groups had one acupuncture treatment during the first 48 hours of menses each cycle.
