# Corrigendum to “More Than Needles: The Importance of Explanations and Self-Care Advice in Treating Primary Dysmenorrhea with Acupuncture”

**DOI:** 10.1155/2018/8468376

**Published:** 2018-04-30

**Authors:** Michael Armour, Hannah G. Dahlen, Caroline A. Smith

**Affiliations:** ^1^The National Institute of Complementary Medicine, Western Sydney University, Locked Bag 1797, Penrith, NSW 2751, Australia; ^2^School of Nursing and Midwifery, Western Sydney University, Locked Bag 1797, Penrith, NSW 2751, Australia

In the article titled “More Than Needles: The Importance of Explanations and Self-Care Advice in Treating Primary Dysmenorrhea with Acupuncture” [1], the Competing Interests section should read as follows:

Michael Armour is a Director of Women's Health and Head of Acupuncture at The Body Workshop Ltd. in New Zealand, though he is not currently a practitioner there.
